# Supplementary material for: Comprehensive Annotation and Functional Exploration of MicroRNAs in Lettuce
Source: Front Plant Sci. 2021 Dec 24;12:781836. doi: 10.3389/fpls.2021.781836 (PMC8739914; doi:10.3389/fpls.2021.781836)
Supplement: Supplementary file 2 [file Data_Sheet_8.ZIP › GSM2230748.plot/Lsa-miRN1699b_Lsat_1_v5_gn_8_143940.1_2537_TPlot.pdf]

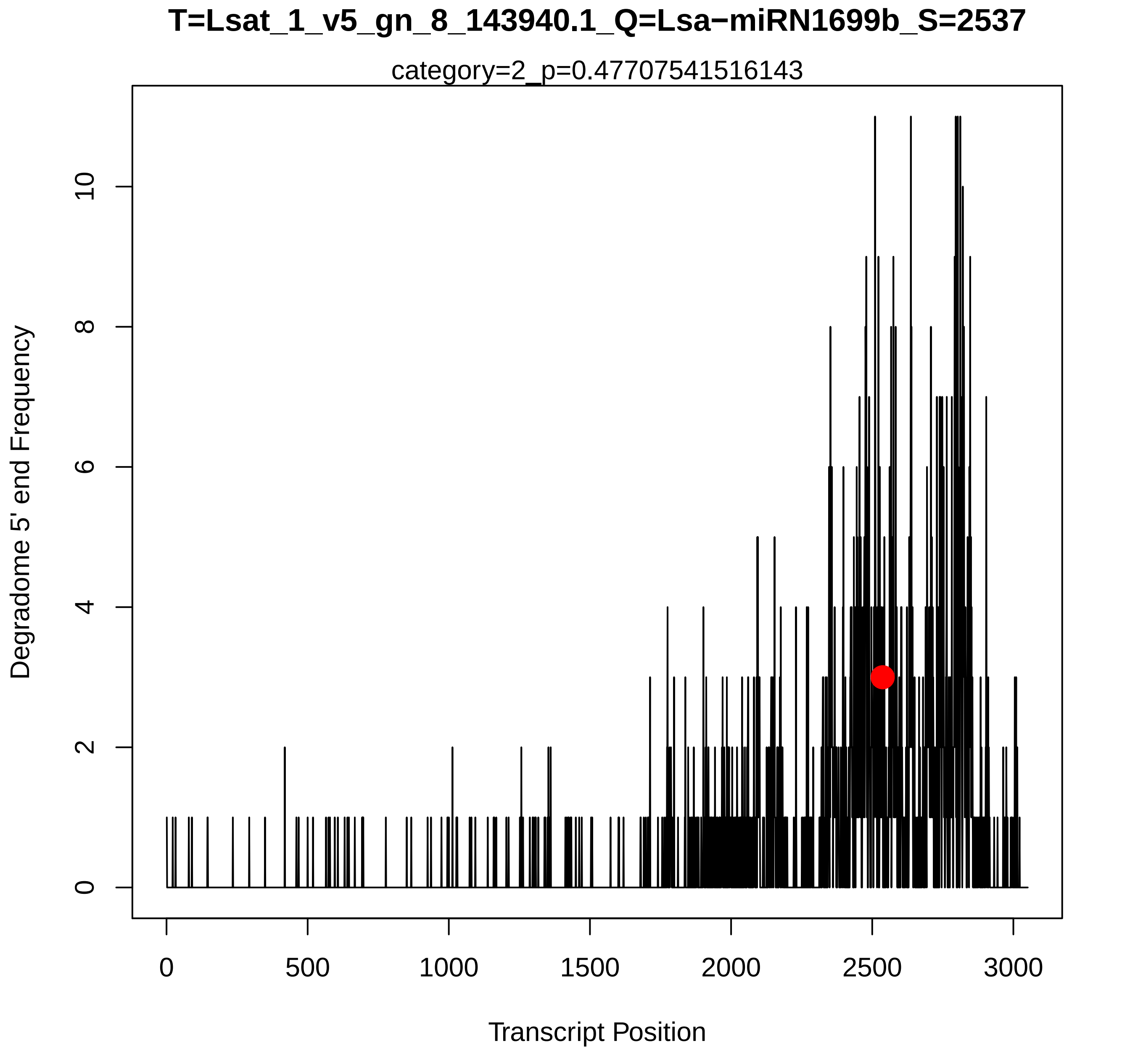

T=Lsat_1_v5_gn_8_143940.1_Q=Lsa−miRN1699b_S=2537
category=2_p=0.477075415161 43
Degradome 5' end Frequency
0
2
4
6
8
10
0
500
1000
1500
2000
2500
3000
Transcript Position
